# Expression of Modified Snowdrop Lectin (*Galanthus nivalis* Agglutinin) Protein Confers Aphids and *Plutella xylostella* Resistance in *Arabidopsis* and Cotton

**DOI:** 10.3390/genes13071169

**Published:** 2022-06-29

**Authors:** Peng He, Huanhuan Jia, Hui Xue, Yuechen Zeng, Lili Tian, Xiaoli Hu, Shufen Chang, Yanli Jiang, Jianing Yu

**Affiliations:** 1 College of Life Sciences, Shaanxi Normal University, Xi’an 710119, China; phe@snnu.edu.cn (P.H.); jiahh0718@163.com (H.J.); xuehui202111@163.com (H.X.); zyc1397cotton@163.com (Y.Z.); mimi178606250@163.com (L.T.); csf2092813@126.com (S.C.); 2 Institute of Cotton Research, Shanxi Agricultural University, Yuncheng 044000, China; huxiaoli209@126.com (X.H.); jylsx@sina.com (Y.J.)

**Keywords:** *Galanthus nivalis* agglutinin, ASGNA, aphids, *Plutella xylostella*, cotton

## Abstract

Cotton is a major fiber crop in the world that can be severely infested by pests in agricultural fields. Identifying new insect-resistance genes and increasing the expression of known insect-resistance genes are imperative in cultivated cotton. *Galanthus nivalis* agglutinin (GNA), a lectin that is toxic to both chewing and sucking pests, is mainly expressed in monocotyledons. It is necessary to improve the expression of the GNA protein and to test whether the lectin confers insect resistance to dicotyledons plants. We report a modified *GNA* gene (*ASGNA*) via codon optimization, its insertion into *Arabidopsis thaliana*, and transient expression in cotton to test its efficacy as an insect-resistance gene against cotton aphids and *Plutella xylostella*. The amount of ASGNA in transgenic plants reached approximately 6.5 μg/g of fresh weight. A feeding bioassay showed that the survival rate of aphids feeding on the leaves of ASGNA transgenic plants was lower than those of aphids feeding on the leaves of non-optimized GNA (NOGNA) transgenic plants and wild-type plants. Meanwhile, the fertility rate was 36% when fed on the ASGNA transgenic plants, while the fertility was 70% and 95% in NOGNA transgenic plants and wild-type plants. Correspondingly, the highest mortality of 55% was found in ASGNA transgenic lines, while only 35% and 20% mortality was observed in NOGNA transgenic plants and wild-type plants, respectively. Similar results were recorded for aphids feeding on cotton cotyledons with transient expression of ASGNA. Taken together, the results show that *ASGNA* exhibited high insecticidal activity towards sap-sucking insects and thus is a promising candidate gene for improving insect resistance in cotton and other dicotyledonous plants.

## 1. Introduction

Cotton is one of the most important crops for fiber and oil and thus is an economically important crop worldwide. Cotton plants are susceptible to damage by pests and diseases, which decrease yield and quality, resulting in economic losses [1,2,3]. Insect pests are a major obstacle to cotton production in many cotton-producing countries, which can contribute to losses of up to 80% [4]. For sustainable fiber production and to minimize the damage caused by a diversity of pests, it is essential to develop varieties that can withstand the ravages of various insect pests. Cotton bollworm (*Helicoverpa armigera*), cotton aphid (*Aphis gossypii,* Glover), green mirid bug (*Apolygus lucorum*), and diamondback moth (*P. xylostella*) are the four major cotton pests that cause severe damage in Chinese fields [5]. These insects directly damage cotton plants by eating plants or sucking the sap with their mouthparts [6,7,8]. Traditionally, chemical pesticides are used to control these pests; however, they cause a number of problems, such as environmental pollution, pesticide residue, and increased production costs [9,10]. Therefore, cultivating quality cotton with high resistance to herbivory is a constant goal for breeders. Transgenic technology offers unique opportunities for controlling pests using gene-engineering technologies to transfer exogenous insecticidal genes into cotton to obtain transgenic insect-resistant cotton [11,12].

Recently, various insecticidal toxin genes from the bacterium *Bacillus thuringiensis* (Bt) and trypsin inhibitors (e.g., cowpea trypsin inhibitor, CpTI), plant lectins, and ribosome-inactivating proteins have been found to be very effective against cotton pests [13,14,15,16,17]. Expressing toxin proteins in crops has shown significant cost savings in terms of time, labor, money, and a reduction in damage to the human population and environment in comparison to traditional chemical insecticides [18,19]. The successful transfer of Bt and other toxin genes into crops, and their effect on enhanced pest resistance has been reported in rice, maize, and cotton [20,21,22]. However, the use of insecticidal genes is not without its limitations: target pests can evolve resistance to these genetically-engineered defenses [23,24,25]. To minimize the problem of insect adaptation to current insecticidal genes, modifying existing toxin genes or pyramiding various resistance genes against target organisms have found some success. Examples of multi-gene pyramids include fusing multiple Bt toxin genes together, linking Bt toxin with other toxin genes, or linking multiple toxin genes in addition to Bt [26,27]. Transgenic upland cotton crops harboring two insecticidal genes, Bt + CpTI or Bt + GNA have been reported to enhance plant resistance to cotton bollworms [28,29].

Cotton aphid, *A. gossypii*, severely affects the growth, yield, and fiber quality of cotton, and also transmits viral diseases and can develop resistance to insecticides [30,31]. Mannose-binding lectin (MBL), a collectin family of proteins with a C-terminal lectin domain including *Galanthus nivalis* agglutinin (GNA), *Allium sativum* agglutinin (ASA), and *Narcissus pseudonarcissus* lectin (NPL), plays a crucial role in plant protection against sap-sucking insects [32,33]. GNA was originally isolated from snowdrop bulbs with specificity towards Manα (1–3) Man-containing oligosaccharides to confer protection against different sap-sucking pests. GNA is a 50 kDa homotetramer that is composed of four non-covalently associated monomers, which can bind to chitin in the peritrophic matrix or by interacting with glycoproteins on the epithelial cells of the insect midgut [34]. As a result, the digestion and assimilation of nutrients are presumably reduced, causing starvation [33,34]. Therefore, GNA is a good candidate gene for improving insect resistance in cotton. However, the sequence of the *GNA* gene from the monocotyledonous plant *G. nivalis* shows a codon usage bias towards monocots; thus, transgenic dicots (e.g., tobacco) exhibit low expression levels of GNA [35]. Therefore, modification of the sequence is likely necessary to increase its protein expression levels to control cotton aphid infestations more efficiently.

We carried out multiple experiments using two insect pests and two different plant species. First, the coding sequence of *GNA* was modified to change very-low-usage bias codons to higher-usage bias ones for improvement of the gene expression in dicot plants by overlapping polymerase chain reaction (PCR). Then the *ASGNA* and the *NOGNA* gene were separately transferred into *A. thaliana* by *Agrobacterium*-mediated transformation. The transgenic plants expressed higher levels of GNA toxin protein in leaf and root tissues. A bioassay of plant resistance to aphids was carried out under artificial conditions, and the results showed that the survival and reproductive rates of aphids were reduced in the ASGNA transgenic group. Additionally, a similar bioassay of plant resistance to *P. xylostella* showed that the weight of *P. xylostella* individuals was reduced to a large extent by ASGNA transgenics. Lastly, we conducted a pest resistance experiment employing transient expression of ASGNA protein in cotton. The results showed that aphid survival and reproductive rates noticeably decreased in aphids exposed to the cotton with a transient expression of the ASGNA protein. The present study shows that the ASGNA protein is toxic to aphids and other herbivorous pests, thus demonstrating the potential of using ASGNA for pest control in cotton.

## 2. Materials and Methods

### 2.1. Plant Materials

*A. thaliana* ecotype Columbia-0 (wild-type) was ordered from ABRC (Ohio State University), and the seeds were planted on agar plates containing Murashige and Skoog salts, 1% Suc (*w*/*v*), and 0.7% (*w*/*v*) agar, and adjusted to a pH of 5.7. After vernalization at 4 °C for at least two days, the seedlings were grown in an illuminated growth chamber at 23 °C. Germinated seedlings were used for *Agrobacterium*-mediated transformation, and the putative transgenic plants were then transferred to pots containing soil of equal proportions of clay, sand, and peat moss (1:1:1). Finally, the plants were moved to a greenhouse and subjected to various molecular analyses and insect bioassays.

The *Gossypium hirsutum* (Xuzhou 142) seeds used in this study were acquired from the Institute of Cotton Research of the Chinese Academy of Agricultural Sciences (Anyang, China). The seeds were planted in containers of sand (one seedling per container) and grown under a 16-h light and 8-h dark cycle at 30 °C in a climate-controlled greenhouse located at Shaanxi Normal University.

### 2.2. Synthesized ASGNA and Transgenic Plants

The original *GNA* sequence was obtained from the National Center for Biotechnology Information (GenBank: M55556.1). In order to optimize the codons of the *GNA* gene to produce ASGNA, a modified SOE-PCR, based on the 16 nt complementary overlapping sequences, was generated to obtain ASGNA using six pairs (F1/R1, F2/R2, F3/R3, F4/R4, F5/R5, F6/R6) of designed primers. The primer sequences used in this study are listed in Supplementary Table S1. A PCR cycler program was performed as follows: 95 °C for 3 min, denaturation at 94 °C for 30 s, annealing at 55 °C for 30 s, and elongation at 72 °C for 1 min. The PCR amplification products were electrophoresed on a 1% agarose gel and purified using the E.Z.N.ATM Gel Extraction Kit (OMEGA Bio-Tek, Norcross, GA, USA). The final SOE (splicing with overlap extension) PCR product was cloned into pMD 19-T Vector (Takara, Nojihigashi, Japan) to obtain ASGNA. The fragment of *NOGNA* was amplified from the pBI121-CaMV35S-GNA plasmid using primer pairs F6/R6 [36]. The primer sequences used for this study are listed in Supplementary Table S1. For stable transformation of *Arabidopsis*, the *ASGNA* gene was cloned into the plant expression vector pART27 to generate pART27-35S:: ASGNA. For transient expression assay, the ASGNA gene was cloned into the pART27-GFP vector to generate pART27-35S::ASGNA-GFP. The pART27-35S::ASGNA-GFP and pART27-35S::ASGNA-GFP constructs were separately introduced into *Agrobacterium tumefaciens* strain GV3101. The pART27-35S::ASGNA constructs were transformed into *A. thaliana* using the *A. tumefaciens* floral-dip procedure [37], and transgenic plants were screened using 50 mg/mL kanamycin.

### 2.3. DNA Isolation

DNA was isolated using the improved CTAB method. *Arabidopsis* plant leaves (0.1 g) were ground into powder in liquid nitrogen. Then, 0.6 mL CTAB extraction buffer was added, and the lysate was incubated at 65 °C for 30 min. The DNA was purified by adding an equal volume of a mixture of chloroform: isoamyl alcohol (24:1) followed by centrifugation at 8000× *g* for 10 min at 4 °C. The supernatant was mixed with a 0.6 volume of isopropanol and then subjected to centrifugation at 8000× *g*. The precipitate was washed twice with 75% ethanol and then dissolved in 300 μL sterile water. We then added NaAc (1/10 volume of 3 M, pH 5.2) and two volumes of ethanol to the dissolved precipitate and incubated each sample for 10 min at −20 °C. The tube was centrifuged at 8000× *g* for 5 min, and the pellet was washed twice with 75% ethanol and re-dissolved in sterile water.

### 2.4. Determination of ASGNA Expression Levels in Transgenic Plants

The total protein was extracted from different tissues (leaf, stem petal, root, and silique) of T1 transgenic plants. Ground leaves were put in 1.5 mL microtubes with 400 μL of protein extraction buffer (150 mM NaCl, 5 mM MgCl_2_, 5 mM DTT, 0.1% NP40, 50 mM, Tris-HCl (pH 7.5), and EDTA-free complete protease inhibitor cocktail). The samples were vortexed to homogenize each sample, incubated at 4 °C for 2 h, and then centrifuged at 13,000× *g* for 10 min. The supernatant was eluted and stored in new 1.5 mL tubes, and Bradford reagent was used to quantify proteins. GNA protein expression was detected using an ELISA kit (SEKH-0316, Meuxuan Biotechnology, Shanghai, China). In brief, 25 μL of either plant extract or purified GNA toxin standards (to obtain final concentrations from 5 to 60 ng of GNA sample in extract buffer) in ELISA plates coated with an anti-GNA antibody were incubated at 37 °C for 2 h in an airtight container with 25 μL of alkaline phosphatase enzyme conjugate. Unbound proteins were removed by washing with phosphate-buffered saline and 0.01% Tween 20 (*v*/*v*). The wells were washed again, and then the assay was developed by the addition of 50 μL p-nitrophenyl phosphate substrate solution, and absorbance was read at 400 nm in a microtiter plate reader (Epoch, BioTek, Winooski, VT, USA). The GNA levels were determined using the GNA calibration curve. Each sample was assayed in triplicate.

### 2.5. Insect Bioassay

Aphid nymphs and *P. xylostella* were collected from the Shaanxi Normal University field station (N, 34°17′, E, 108°93′, Shaanxi Province, Northwest China) in July and grown under laboratory conditions for a feeding bioassay. To investigate whether transgenic plants expressing ASGNA could confer enhanced resistance to *P. xylostella*, insect larvae were fed either on the leaves of the control or T1 transgenic *Arabidopsis* plants (45-day-old) in Petri-dishes, with one larva of each species per dish. To investigate the aphid resistance of ASGNA plants, the leaves of the control or T1 transgenic *Arabidopsis* plants were confined in an insect-proof fine-mesh nylon cage, and 10 late-instar aphid nymphs were introduced with a hairbrush to plant leaves of each plant on Day 0. Survival and growth rates of the insect populations were determined at 2-day intervals for a 14-day period. The cocoon proportion was calculated as follows: (number of larva with cocoon/number of total larvae) × 100%. All of the experiments were repeated three times (each independently derived transgenic line was micropropagated into three cloned plants).

### 2.6. Transient GNA Expression in Cotton

For the transient expression assay, the full-length amino acid sequences of NOGNA or ASGNA were fused to the N terminus of GFP protein under the control of the 2 × CaMV35S promoters in the transient expression vector pCAMBIA1305-GFP. Cotyledon disks excised from 10–12-day-old cotton seedlings were used for transient expression analysis of GNA. *Agrobacterium* (GV3101) cultures were grown overnight at 28 °C in a lysogeny broth medium containing 50 μg/mL kanamycin, 25 μg/mL gentamicin, 10 mm MES, and 20 μm acetosyringone. The cells were pelleted by centrifugation at 1500× *g* at room temperature for 5 min and resuspended in an infiltration culture containing 10 mm MgCl_2_, 10 mm MES, and 200 μm acetosyringone. Cell suspensions were incubated at room temperature for at least 3 h. *Agrobacterium* (GV3101) cultures containing the GNA expression vector were infiltrated into two fully expanded cotyledons of two-week-old plants using a needle-less syringe. To facilitate the infiltration, small holes were punched with a needle on the underside of the cotyledon. These experiments were repeated at least three times, with more than six plants per replicate.

## 3. Results

### 3.1. PCR Amplification of ASGNA Gene

**Figure 1:**
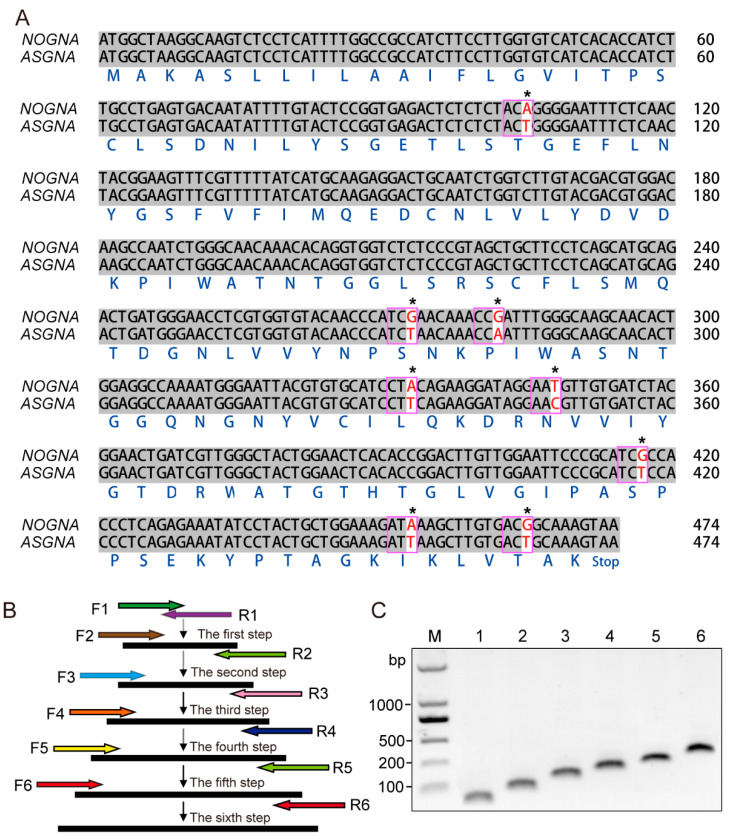
Cloning of *ASGNA* genes. (**A**) Sequence alignment of non-optimized *G. nivalis* agglutinin (NOGNA) with selected codons replaced to modify *GNA* (*ASGNA*). Numbers flanking the sequences are nucleotide positions. The replaced nucleotides are indicated by asterisks (*). The optimized codons are marked with purple box. (**B**) Schematic diagram of the synthesis of the *ASGNA* gene by overlapping PCR. Primer pairs used in PCR amplifications are F1/R1 (step 1), F2/R2 (step 2), F3/R3 (step 3), F4/R4 (step 4), F5/R5 (step 5) and F6/R6 (step 6). (**C**) The amplification of the *ASGNA* gene by six overlapping PCRs. M: Marker. 1–6 are PCR products from step 1 to step 6, respectively.

To optimize the codons and achieve high levels of *GNA* expression in cotton, we analyzed the sequence of *NOGNA* and found that eight codons were infrequently used in dicotyledons (Figure 1A). We replaced these eight codons with frequently used dicotyledonous codons based on codon usage bias without changing the amino acids. After six PCRs amplification, we obtained a 474-bp ASGNA fragment (Figure 1B,C), and the sequencing results confirmed that the codons were successfully replaced.

### 3.2. Expression of ASGNA in Transgenic A. thaliana

**Figure 2:**
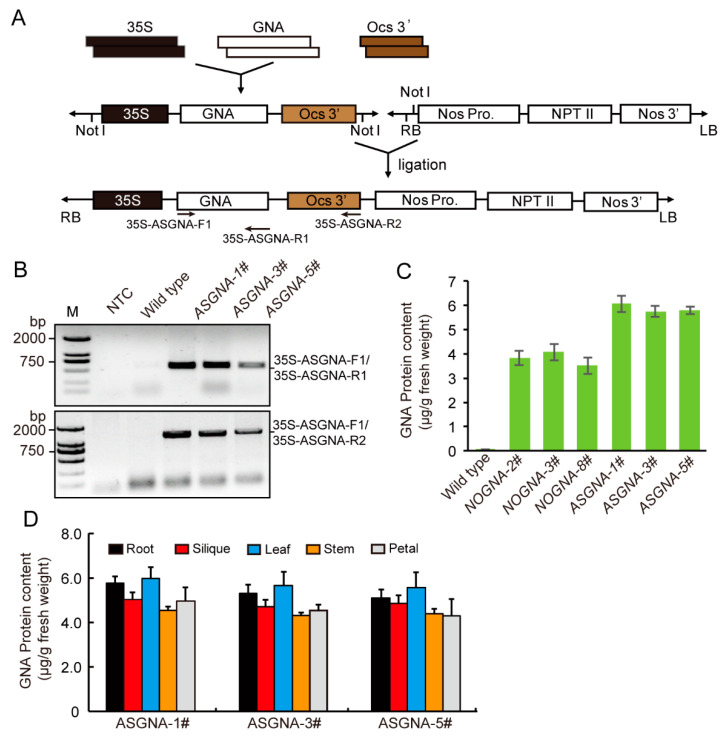
Generation and confirmation of ASGNA transgenic plants. (**A**) Schematic representation of the CaMV35S-ASGNA expression construct in a plant transformation vector. 35S, CaMV35S promoter. NPT II, neomycin phosphotransferase II gene. RB, right border. LB, left border. (**B**) Detection of transgenic plants by amplification of *ASGNA* using the genomic DNA template. Upper, the ASGNA fragment was amplified using primers 35S-ASGNA-F1 and 35S-ASGNA-R1. Lower, the ASGNA and Ocs fragment was amplified using primers 35S-ASGNA-F1 and 35S-ASGNA-R2. The primers used in PCR amplification are shown in (**A**). Lane M, DNA marker DL2000. Lane “NTC”, no-template control. (**C**) GNA content in wild-type, NOGNA transgenic lines, and ASGNA transgenic lines. ELISA was performed to measure the total protein extracted from seedling of transgenic lines. Three replicate assays were set up using a single crude extract from plant tissues. (**D**) Protein expression of ASGNA in transgenic lines. ELISA was performed to measure the total protein extracted from different tissues of transgenic lines.

The full-length ASGNA fragment was cloned into a plant expression vector under the control of a constitutively modified CaMV 35S promoter (Figure 2A). The resultant construct was transformed into *Arabidopsis* plants. After the selection of T0 seeds with kanamycin, ASGNA transformation was successfully achieved in several independent lines. The independently generated transgenic lines were analyzed using PCR amplification to confirm the transformation. As is shown in Figure 2B, a 474-bp fragment was only amplified from the genome of transgenic plants using primers 35S-ASGNA-F1 and 35S-ASGNA-R1, and a 1582-bp fragment was only amplified from the genome of transgenic plants using primers 35S-ASGNA-F1 and 35S-ASGNA-R2, indicating that the ASGNA gene had successfully integrated into *Arabidopsis* genomic DNA (Figure 2B). Three homozygous T1 transgenic plants of *NOGNA* and ASGNA were selected through Mendelian segregation, and their progeny were used for subsequent experiments. We performed an enzyme-linked immunosorbent assay (ELISA) assay to detect the protein expression of GNA in NOGNA and ASGNA transgenic lines. The results showed that the three ASGNA transgenic lines had similar expression levels of ASGNA, of which the GNA protein content was approximately 6 μg/g fresh weight. We found that the GNA protein contents of the three ASGNA transgenic lines are much higher than that of the *NOGNA* transgenic lines (Figure 2C). In addition, the leaves of ASGNA transgenic line 1 (ASGNA-1#) exhibited higher GNA protein levels than those of the other tissues (Figure 2D). Thus, the leaves of ASGNA-1# were used for further experiments.

### 3.3. Effects of ASGNA Toxin on Aphids

**Figure 3:**
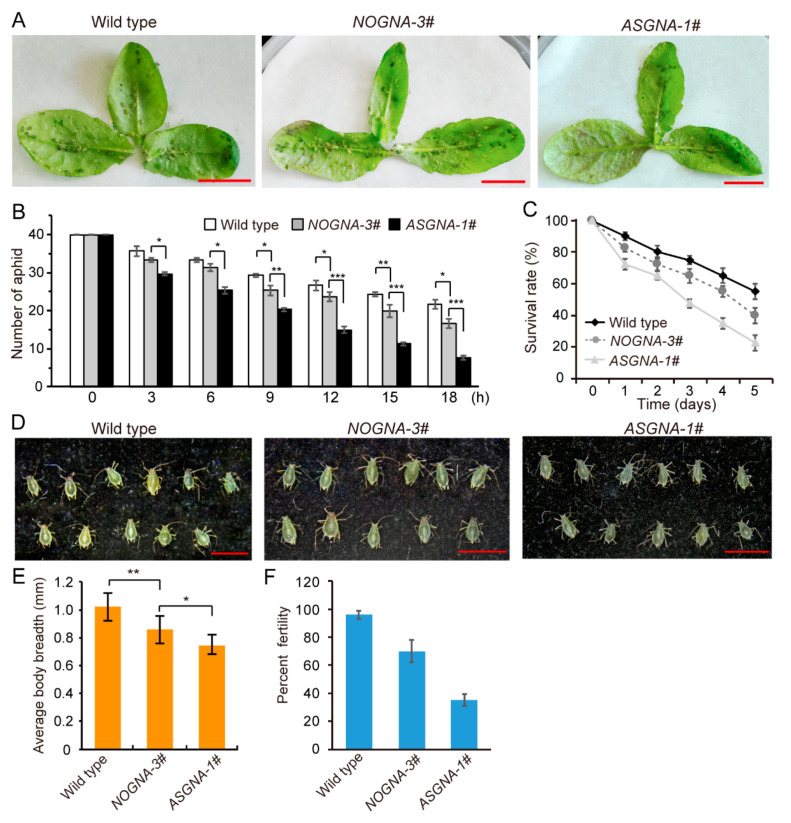
Aphid feeding bioassay of wild-type and transgenic lines. (**A**) *Arabidopsis* leaves were fed to aphids. Bars = 1.0 cm. (**B**) The number of aphids feeding on transgenic plants expressing *ASGNA* reduced over time. Forty individuals were used in each of three replications. The significance differences between transgenic plants and wild-type plants were determined from the independent samples; the error bars represent SE of the means. *, *p* < 0.05. **, *p* < 0.01. ***, *p* < 0.001, Student’s *t*-test. (**C**) Survival rate of aphids fed on different plant lines c. Each experiment was performed with three biological replicates, and the error bars represent SE of the means. (**D**) Morphological characteristics of aphids fed wild-type or transgenic leaves. Bars = 2 mm. (**E**,**F**) Body breadth and percent fertility analysis of aphids fed on different plant lines. Each experiment was performed with three biological replicates, and the error bars represent SE of the means. *, *p* < 0.05. **, *p* < 0.01. Student’s *t*-test.

In order to test whether the modified ASGNA lectin confers insect resistance for dicotyledons, a bioassay with *Aphis gossypii* (*A. gossypii*) was performed with the transgenic *Arabidopsis*. Second-instar aphids were transferred to fresh detached *Arabidopsis* leaves (Figure 3A), and after 18 h of feeding, we observed that the number of aphids on ASGNA transgenic *Arabidopsis* plants was less than that on *NOGNA* transgenic plants and wild-type plants, indicating that aphids were likely to avoid plants of the transgenic line expressing ASGNA (Figure 3B). In the survival analysis, great differences in survival curves were found between the ASGNA transgenics and the *NOGNA* transgenics (Figure 3C). Moreover, we noticed that the reproductive rate and body width of surviving aphids on the ASGNA plants were affected in comparison to those of the *NOGNA* transgenic group and the wild-type group (Figure 3D,E). In wild-type plants and *NOGNA* transgenic plants, the body width of surviving aphids was approximately 1.02 mm and 0.85 mm, respectively. Furthermore, the body breadth of aphids was reduced to 0.74 mm in ASGNA transgenic plants (Figure 3E). Statistical analysis of the fertility rate showed that aphids on *NOGNA* transgenic plants were nearly 70%, whereas, in ASGNA transgenic lines, the rate was reduced to approximately 36% (Figure 3F). These results indicate that the modified ASGNAs have a strong antipest toxin that affects aphids, which confers insect resistance to dicotyledon plants.

### 3.4. Effects of ASGNA Toxin on P. xylostella

**Figure 4:**
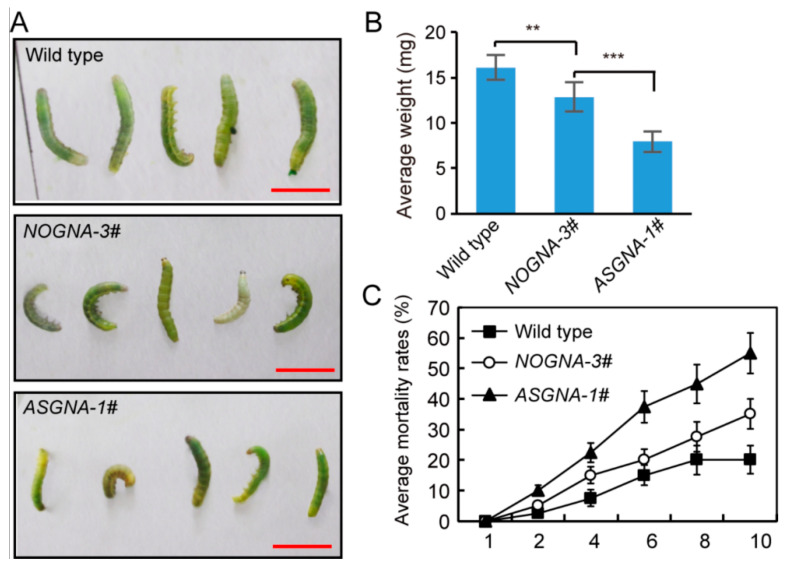
Effect of transgenic *Arabidopsis* lines expressing ASGNA on *P.* *xylostella* larvae. (**A**) The body characteristics of larvae fed on different plant lines, including *ASGNA, NOGNA,* and wild-type control. The photo was taken 7 days after feeding. Bars = 1 cm. (**B**) The weight of larvae (7 days of feeding) fed with transgenic plants expressing ASGNA significantly decreased. Error bars indicate standard errors of means of three biological replicates. **, *p* < 0.01. ***, *p* < 0.001, Student’s *t*-test. (**C**) Larval mortality rates of individuals fed on *ASGNA* transgenic leaves was obviously increased compared to that of *NOGNA* and wild-type control.

*P. xylostella* is also an important cotton pest that is difficult to control. To test the efficacy of GNA proteins expressed in plants against *P. xylostella*, a bioassay with *P.* *xylostella* was performed using transgenic *Arabidopsis*. *P.* *xylostella* larvae were placed on the leaves of the transgenic plants and wild-type plants, respectively. Compared to *NOGNA* and wild-type controls, *P.* *xylostella* that fed on ASGNA transgenic plants was significantly impaired, and they had smaller body sizes (Figure 4A). Larval weights were also reduced to a large extent seven days after the start of the experiment. The larvae on ASGNA transgenic plants weighed approximately 8 mg, while the larvae on wild-type plants weighed approximately 15 mg and 12 mg on *NOGNA* plants (Figure 4B). The highest mortality of ASGNA transgenic lines was observed, ranging from 48.3% to 61.6%, while the mortality of the *NOGNA* controls was lower, ranging from 30.1% to 39.9%. The average mortality rates from the ASGNA transgenic, *NOGNA* transgenic, and wild-type groups were 55.0%, 35.0%, and 20%, respectively (Figure 4C). These results indicate that the resistance levels of transgenic lines containing ASGNA were higher than those in NOGNA and wild-type controls.

### 3.5. Transient Expression of ASGNA in Cotton Cotyledons Resulted in Enhanced Resistance to Aphids

**Figure 5:**
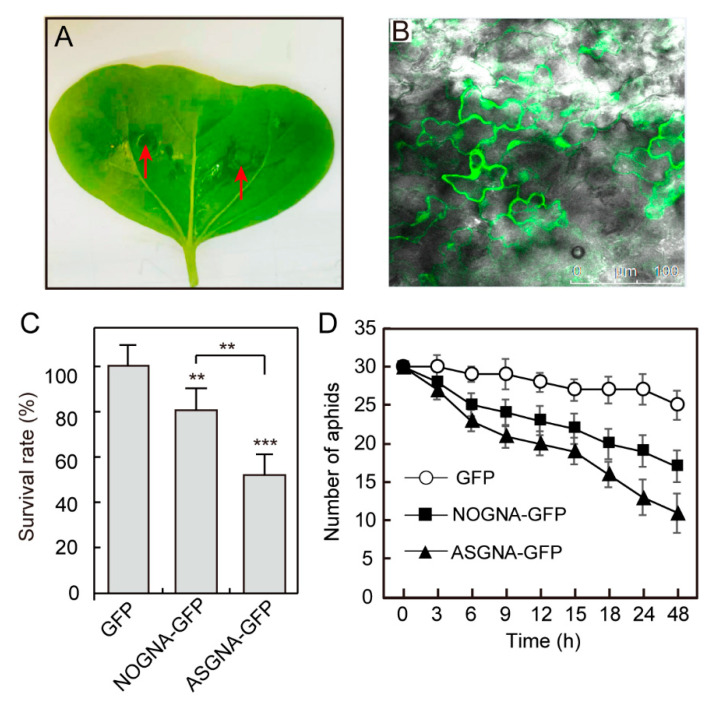
Analysis of aphid resistance and the transient expression of ASGNA in cotton cotyledons. (**A**) Full of each cotton cotyledon was injected for transient expression of ASGNA. The red arrow depicts the injection point. (**B**) Protein expression was detected by GFP fluorescence. (**C**) Survival rates of aphids significantly decreased after feeding on transiently-expressed ASGNA cotton cotyledons. Each experiment was performed in three biological replicates, and the error bars represent SEs of the means. **, *p* < 0.01, ***, *p* < 0.001, Student’s *t*-test. (**D**) The number of aphids was reduced after feeding on cotyledons expressing ASGNA. The error bars represent SEs of the means.

Transient gene expression is an attractive alternative method that allows transgenes to be assayed more rapidly and easily, so we conducted a transient transformation in cotton cotyledons to test for insect resistance (Figure 5A). To observe the protein expression, a green fluorescent protein (*GFP*) gene was introduced and fused with NOGNA or ASGNA to construct a recombinant fusion protein. The *Agrobacterium* cultures containing NOGNA-GFP or the ASGNA-GFP expression vector were injected into cotton cotyledons. We detected strong GFP fluorescence after three days, indicating that NOGNA and ASGNA were expressed in cotton cotyledons (Figure 5B).

The bioassay using second-instar aphids to feed on cotton cotyledons for 48 h showed that the survival rate of aphids on transiently-expressed-ASGNA cotyledons was lower than that of the transiently-expressed-NOGNA group and transiently-expressed-GFP group (Figure 5C). The number of aphids was also counted two days after their transfer to the cotyledons. We found that aphids exposed to transiently-expressed-ASGNA cotyledons showed a significant decrease in populations when compared with that of the transiently-expressed-NOGNA group and transiently-expressed-GFP group (Figure 5D). Moreover, the aphid populations on transiently-expressed-ASGNA cotyledons were reduced by 50% when compared with that of the transiently-expressed-GFP group after two days of feeding, showing that the transient expression of ASGNA in cotyledons had inhibitory effects on the growth and reproduction of aphids.

## 4. Discussion

In the past, strategies aimed at reducing crop losses relied primarily on chemical pesticides. However, the long-term use of chemical pesticides has caused ecological destruction and increased pesticide resistance in insect pests. Transgenic plants conferring pest resistance would be an alternative tool for pest management. Because some pests have evolved resistance to the toxic proteins that confer resistance to pests in transgenic cotton [38], new and more effective insect-resistance genes need to be developed.

Plant lectins are non-immune origin glycoproteins that selectively bind to carbohydrates, agglutinate cells, or precipitate polysaccharides and glycoconjugates [39,40,41]. Recent advances in our understanding of the biochemical and molecular mechanisms of plant lectins have been provided by research focused on GNA-related lectins. Snowdrop (*G. nivalis*), a type of (GNA)-related ectinfamily, was exclusively isolated from a subgroup of monocotyledonous plants in 1987 [42]. Considering that GNA is relatively safe for mammals because it lacks the appropriate lectin receptors, the encoded gene of GNA has been cloned and modified to confer pest resistance in plants via genetic engineering since snowdrop lectin was first isolated. Yuan et al. (2001) modified nine sites of the GNA coding sequence by site-directed mutagenesis based on codon usage bias to improve the gene expression of lectin in *Nicotiana tabacum* [35]. Western blot analysis showed that the expression level of GNA in GNA34m (modified GNA) transgenic tobacco plants was 0.25% of the total soluble proteins and was much higher than that in GNA34 (non-modified GNA) transgenic plants. Moreover, the GNA34m lines exhibited greater aphid resistance when compared to that of GNA34 transgenic lines. In our study, eight sites of the GNA coding sequence were modified with respect to the dicot-biased codon usage. Among the eight sites, four are in common with Yuan’s study. The ASGNA gene was transformed into *Arabidopsis* plants, and GNA protein levels in transgenic plants were approximately 6 μg/g fresh weight. In addition, there were no significant differences in ASGNA protein content among the three transgenic lines. Previous studies have reported that the expression of GNA protein constituted 0.4% of the total soluble protein in transgenic lettuce and 0.3% to 0.4% of the total soluble protein in transgenic potato [36,43]. In this study, the GNA content accounted for 0.41–0.47% of the total soluble protein, which was slightly higher than previously reported in dicotyledons. We suspect that our synthetic ASGNA gene, which was based on the preferred codons of dicotyledons, was more suitable for expression in dicotyledons. Rao et al. reported that the level of GNA protein expression was up to 2.0% of the total soluble protein in transgenic rice plants using a phloem-specific promoter [44]. Further experiments will be required to determine the levels of ASGNA protein expression derived by phloem-specific promoters in the next work.

The toxicity level of GNA lectin may vary according to the type and age of the insect, as well as the type of animal. For instance, GNA has a moderate level of toxicity to insect pests with chewing mouthparts (e.g., Lepidoptera, Diptera) and a significant level of toxicity to insect pests with piercing-sucking mouthparts (e.g., aphids, brown planthoppers, and nematodes) [45]. Thus, GNA is valuable for improving insect resistance in plants while reducing potential harm to humans and other non-target animals. In potato, GNA expressed under the CaMV 35S promoter confers some resistance to aphid attack; the aphid population exposed to transgenic plants ranged from 24.9 to 53.5% of the aphid population exposed to non-transformed plants. Yuan et al. (2001) constructed a plant expression vector with cloned GNA, a double CaMV 35S enhancer, and a CoYMV promoter with tissue-specific expression in the phloem [35]. Then, tobacco plants were transformed with the construct. The transformed tobacco plants exhibited strong anti-aphid activity, which reduced the aphid population by 45–60% on average [43]. Li and Romeis (2009) fed lacewing flies with an artificial diet containing different concentrations of GNA and found that GNA not only delayed the development of lacewing larvae and reduced longevity, daily fecundity, and total fecundity of adults [46]. In our bioassay of transgenic *Arabidopsis*, the aphid population on transgenic plants was less than 50% of the population in control plants, indicating that the transgenic *Arabidopsis* had greater resistance to aphids. Furthermore, the average aphid population of transiently-expressed-ASGNA cotyledons was reduced to about half that of wild-type rates, indicating that ASGNA is toxic to piercing–sucking insect pests. Therefore, ASGNA is a viable candidate gene for conferring pest resistance to cotton aphids in cotton breeding. Chewing insect pests also cause damage to cotton. Transgenic tobacco plants expressing the GNA protein show increased resistance and toxicity to one chewing pest, the cotton bollworm, *Helicoverpa armigera*. Sétamou et al. found that larval survival, percent adult emergence, and female fecundity of Eoreuma loftini were significantly reduced when fed with transgenic GNA sugarcane. In potato, transgenic plants expressing GNA can significantly reduce the survival rate of *Lacanobia oleracea* larval by approx. 40%. In our study, we found that larval weights of *P.* *xylostella* were reduced to a large extent, and the mortality of *P.* *xylostella* was increased when fed on the ASGNA transgenic plants. Hence, ASGNA molecules could be used in insect management alone or along with available entomotoxic molecules to overcome agricultural insects and pests.

Nontoxicity to non-target insects is very important for agricultural applications of insect-resistant genes. Phytolectin is a mannose-binding lectin found in monocotyledons and has strong similarities to GNA in amino acid sequences, binding specificity, and molecular structure. Peumans et al. (1997) fed leek flowers that highly expressed phytolectin to the non-target insect bees and found that the phytolectin lost its activity during the conversion of nectar into honey [47]. Down et al. (2000) found that transgenic plants expressing GNA had no obvious effects on the development and survival of *Adalia bipunctata* larvae that preyed on the aphids fed with these transgenic plants [48]. These results suggest that GNA may be non-toxic to small predators of aphids and other pests. However, whether or not the synthesized ASGNA gene has deleterious effects on beneficial insects remains to be further studied.

## 5. Conclusions

From the present study, it is interesting to note that the modified ASGNA can be highly expressed in dicotyledons *Arabidopsis*. The ASGNA transgenic plants showed high insecticidal activity towards sap-sucking insects, and the transient expression of ASGNA in cotton cotyledons also showed enhanced resistance to aphids. In addition, the expression of ASGNA in *Arabidopsis* was effective against the chewing insects. All of the results indicate that expressing the ASGNA protein in transgenic plants could be a useful approach for controlling sap-sucking and chewing pests in dicotyledon plants in the future. Hence, the present findings of ASGNA could be considered valuable entomotoxic molecules against insect pests and appear to be a promising resource in conventional breeding to develop transgenic plants resistant to agriculture pests.

## Data Availability

The data presented in this study are available on request from the corresponding author.

## Supplementary Materials

The following supporting information can be downloaded at: https://www.mdpi.com/article/10.3390/genes13071169/s1, Table S1: A list of primers used in this study.

## References

[1] Luo S.P., Naranjo S.E., Wu K.M. (2014). Biological control of cotton pests in China. Biol. Control.

[2] Wu K.M., Guo Y.Y. (2005). The evolution of cotton pest management practices in China. Annu. Rev. Entomol..

[3] Bestete L.R., Torres J.B., Silva R.B.B., Silva-Torres C.S.A., Bastos C.S. (2017). Development of cotton pests exhibiting different feeding strategy on water-stressed and kaolin-treated cotton plants. J. Pest Sci..

[4] Llandres A.L., Almohamad R., Brevault T., Renou A., Tereta I., Jean J., Goebel F.R. (2018). Plant training for induced defense against insect pests: A promising tool for integrated pest management in cotton. Pest Manag. Sci..

[5] Chen D., Chen F., Chen C., Chen X., Mao Y. (2017). Transcriptome analysis of three cotton pests reveals features of gene expressions in the mesophyll feeder *Apolygus lucorum*. Sci. China Life Sci..

[6] Wilson L.J., Whitehouse M.E.A., Herron G.A. (2018). The management of insect pests in Australian cotton: An evolving story. Annu. Rev. Entomol..

[7] Wang S.Y., Qi Y.F., Desneux N., Shi X.Y., Biondi A., Gao X.W. (2017). Sublethal and transgenerational effects of short-term and chronic exposures to the neonicotinoid nitenpyram on the cotton aphid *Aphis. gossypii*. J. Pest Sci..

[8] Wu K.M., Lu Y.H., Feng H.Q., Jiang Y.Y., Zhao J.Z. (2008). Suppression of cotton bollworm in multiple crops in china in areas with Bt toxin-containing cotton. Science.

[9] Lacey L.A., Georgis R. (2012). Entomopathogenic nematodes for control of insect pests above and below ground with comments on commercial production. J. Nematol..

[10] Savci S. (2012). An agricultural pollutant: Chemical fertilizer. Int. J. Environ. Sci. Dev..

[11] Tester M., Langridge P. (2010). Breeding technologies to increase crop production in a changing world. Science.

[12] Lu Y.H., Wu K.M., Jiang Y.Y., Xia B., Li P., Feng H.Q., Wyckhuys K.A.G., Guo Y.Y. (2010). Mirid bug outbreaks in multiple crops correlated with wide-scale adoption of Bt cotton in China. Science.

[13] Kathage J., Qaim M. (2012). Economic impacts and impact dynamics of Bt (*Bacillus thuringiensis*) cotton in India. Proc. Natl. Acad. Sci. USA.

[14] Mohan K.S., Ravi K.C., Suresh P.J., Sumerford D., Head G.P. (2016). Field resistance to the *Bacillus thuringiensis* protein Cry1Ac expressed in Bollgard hybrid cotton in pink bollworm, *Pectinophora gossypiella* (Saunders), populations in India. Pest Manag. Sci..

[15] Katara J.L., Kaur S., Kumari G.K., Singh N.K. (2016). Prevalence of *cry2*-type genes in *Bacillus thuringiensis* isolates recovered from diverse habitats in India and isolation of a novel *cry2Af2* gene toxic to *Helicoverpa armigera* (cotton boll worm). Can. J. Microbiol..

[16] Cui J.J., Luo J.Y., Van der Werf W., Ma Y., Xia J.Y. (2011). Effect of pyramiding Bt and CpTI genes on resistance of cotton to *Helicoverpa armigera* (Lepidoptera:Noctuidae) under laboratory and field conditions. J. Econ. Entomol..

[17] Din S.U., Azam S., Rao A.Q., Shad M., Ahmed M., Gul A., Latif A., Ali M.A., Husnain T., Shahid A.A. (2021). Development of broad-spectrum and sustainable resistance in cotton against major insects through the combination of *Bt* and plant *lectin* genes. Plant Cell Rep..

[18] Fourie D., van den Berg J., du Plessis H. (2017). Efficacy of *Bacillus thuringiensis* sprays and cotton cultivars expressing Cry proteins in the control of *Earias biplaga* (Walker) (Lepidoptera: Noctuidae). Afr. Entomol..

[19] Chen Z.H., Wei K., Chen L.J., Wu Z.J., Luo J.Y., Cui J.J. (2017). Effects of the consecutive cultivation and periodic residue incorporation of *Bacillus thuringiensis* (Bt) cotton on soil microbe-mediated enzymatic properties. Agric. Ecosyst. Environ..

[20] Carriere Y., Ellers-Kirk C., Sisterson M., Antilla L., Whitlow M., Dennehy T.J., Tabashnik B.E. (2003). Long-term regional suppression of pink bollworm by *Bacillus thuringiensis* cotton. Proc. Natl. Acad. Sci. USA.

[21] Zhao J.Z., Cao J., Li Y., Collins H.L., Roush R.T., Earle E.D., Shelton A.M. (2003). Transgenic plants expressing two *Bacillus thuringiensis* toxins delay insect resistance evolution. Nat. Biotechnol..

[22] Liu B., Cui J., Meng J., Hu W., Luo J., Zheng Y. (2009). Effects of transgenic Bt+CpTI cotton on the growth and reproduction of earthworm *Eisenia foetida*. Front. Biosci..

[23] Wu H.S., Shi X., Li J., Wu T.Y., Ren Q.Q., Zhang Z.H., Wang M.Y., Shang X.X., Liu Y., Xiao S.H. (2016). Effects of root exudates of bivalent transgenic cotton (Bt+CpTI) plants on antioxidant proteins and growth of conventional cotton (Xinluhan 33). J. Environ. Biol..

[24] Wang P., Zhuo X.R., Tang L., Liu X.S., Wang Y.F., Wang G.X., Yu X.Q., Wang J.L. (2017). C-type lectin interacting with β-integrin enhances hemocytic encapsulation in the cotton bollworm, *Helicoverpa armigera*. Insect Biochem. Mol. Biol..

[25] Vanti G.L., Katageri I.S., Inamdar S.R., Hiremathada V., Swamy B.M. (2018). Potent insect gut binding lectin from *Sclerotium rolfsii* impart resistance to sucking and chewing type insects in cotton. J. Biotechnol..

[26] Ma X.F., Yu C.M., Tang S.W., Guo S.D., Zhang D., Wang Y.Z., Zhu A.G., Zhu S.Y., Xiong H.P. (2010). Transgenic of ramie with synthetic CryIA+CpTI gene by *Agrobacterium tumefaciens*-mediated. Acta Agron. Sin..

[27] Li P., Li Y., Shi J., Yu Z., Pan A., Tang X., Ming F. (2018). Impact of transgenic Cry1Ac + CpTI cotton on diversity and dynamics of rhizosphere bacterial community of different root environments. Sci. Total. Environ..

[28] Guo S., Cui H.Z., Xia L., Wu D.L., Ni W., Zhang Z.L., Zhang B.L., Xu Y.J. (1999). Development of bivalent insect-resistant transgenic cotton plants. Sci. Agric. Sin..

[29] Yao Y.S., Han P., Niu C.Y., Dong Y., Gao X.W., Cui J.J., Desneux N. (2016). Transgenic Bt cotton does not disrupt the top-down forces regulating the cotton aphid in central china. PLoS ONE.

[30] Cao C.-W., Zhang J., Gao X.-W., Liang P., Guo H.-L. (2008). Overexpression of carboxylesterase gene associated with organophosphorous insecticide resistance in cotton aphids, Aphis gossypii (Glover). Pestic. Biochem. Physiol..

[31] Gong Y.-H., Yu X.-R., Shang Q.-L., Shi X.-Y., Gao X.-W. (2014). Oral delivery mediated RNA interference of a carboxylesterase gene results in reduced resistance to organophosphorus insecticides in the cotton Aphid, *Aphis gossypii* Glover. PLoS ONE.

[32] Upadhyay S.K., Mishra M., Singh H., Ranjan A., Chandrashekar K., Verma P.C., Singh P.K., Tuli R. (2010). Interaction of *Allium sativum* leaf agglutinin with midgut brush border membrane vesicles proteins and its stability in *Helicoverpa armigera*. Proteomics.

[33] Upadhyay S.K., Singh P.K. (2012). Receptors of garlic ( *Allium sativum* ) lectins and their role in insecticidal action. Protein J..

[34] Zhu-Salzman K., Shade R.E., Koiwa H., Salzman R.A., Narasimhan M., Bressan R.A., Hasegawa P.M., Murdock L.L. (1998). Carbohydrate binding and resistance to proteolysis control insecticidal activity of *Griffonia simplicifolia* lectin II. Proc. Natl. Acad. Sci. USA.

[35] Yuan Z.Q., Zhao C.Y., Zhou Y., Tian Y.C. (2001). Aphid-resistant transgenic tobacco plants expressing modified *gna* gene. Acta Bot. Sin..

[36] Mi X.X., Liu X., Yan H.L., Liang L.N., Zhou X.Y., Yang J.W., Si H.J., Zhang N. (2017). Expression of the *Galanthus nivalis agglutinin* (GNA) gene in transgenic potato plants confers resistance to aphids. Comptes Rendus Biol..

[37] Clough S.J., Bent A.F. (1998). Floral dip: A simplified method for Agrobacterium-mediated transformation of *Arabidopsis thaliana*. Plant J..

[38] Jin L., Wang J., Guan F., Zhang J., Yu S., Liu S., Xue Y., Li L., Wu S., Wang X. (2018). Dominant point mutation in a tetraspanin gene associated with field-evolved resistance of cotton bollworm to transgenic Bt cotton. Proc. Natl. Acad. Sci. USA.

[39] Goldstein I.J., Hughes R.C., Monsigny M., Osawa T., Sharon N. (1980). What should be called a lectin?. Nature.

[40] Sharon N., Lis H. (1989). Lectins as cell recognition molecules. Science.

[41] Sharon N. (2007). Lectins: Carbohydrate-specific reagents and biological recognition molecules. J. Biol. Chem..

[42] Damme E., Allen A., Peumans W. (1987). Isolation and characterization of a lectin with exclusive specificity towards mannose from snowdrop (*Galanthus nivalis*) bulbs. FEBS Lett..

[43] Lupan I., Valimareanu S., Coste A., Popescu O. (2010). Molecular cloning of agglutinin gene from *Galanthus nivalis* for Lettuce transformation. Rom. Biotechnol. Lett..

[44] Rao K.V., Rathore K.S., Hodges T.K., Fu X., Stoger E., Sudhakar D., Williams S., Christou P., Bharathi M., Bown D.P. (1998). Expression of snowdrop lectin (GNA) in transgenic rice plants confers resistance to rice brown planthopper. Plant J..

[45] Liu S.M., Li J., Zhu J.-Q., Wang X., Wang C.S., Liu S.S., Chen X.X., Li S. (2015). Transgenic plants expressing the AaIT/GNA fusion protein show increased resistance and toxicity to both chewing and sucking pests. Insect Sci..

[46] Li Y., Romeis J. (2009). Impact of snowdrop lectin (*Galanthus nivalis* agglutinin; GNA) on adults of the green lacewing, *Chrysoperla carnea*. J. Insect Physiol..

[47] Peumans W.J., Smeets K., Van Nerum K., Van Leuven F., Van Damme E.J. (1997). Lectin and alliinase are the predominant proteins in nectar from leek (*Allium porrum* L.) flowers. Planta.

[48] Down R., Ford L., Woodhouse S., Raemaekers R., Leitch B., Gatehouse J., Gatehouse A. (2000). Snowdrop lectin (GNA) has no acute toxic effects on a beneficial insect predator, the 2-spot ladybird (*Adalia bipunctata* L.). J. Insect Physiol..

