# Supplementary material for: Expression of Modified Snowdrop Lectin (Galanthus nivalis Agglutinin) Protein Confers Aphids and Plutella xylostella Resistance in Arabidopsis and Cotton
Source: Genes (Basel). 2022 Jun 29;13(7):1169. doi: 10.3390/genes13071169 (PMC9316576; doi:10.3390/genes13071169)
Supplement: Supplementary file 1 [file genes-13-01169-s001.zip › genes-1743446-supplementary.pdf]

Table S1. A list of primers used in this study.

Name	Sequence 5'-3'	Experiment
ASGNA-F1	TGGGCAACAAACACAGGTGGTCTCTCCCGTAG CTGCTTCCTCAGCATGCAGACTGAT	over-lap PCR
ASGNA-R1	AAATTGGTTTGTTAGATGGGTTGTACACCACGA GGTTCCCATCAGTCTGCATGCTGA	
ASGNA-F2	AGAGGACTGCAATCTGGTCTTGTACGACGTGG ACAAGCCAATCTGGGCAACAAACACA	over-lap PCR
ASGNA-R2	ATGCACACGTAATTCCCATTTTGGCCTCCAGTG TTGCTTGCCCAAATTGGTTTGTTAGA	
ASGNA-F3	TGGGGAATTTCTCAACTACGGAAGTTTCGTTTT TATCATGCAAGAGGACTGCAATCT	over-lap PCR
ASGNA-R3	AACGATCAGTTCCGTAGATCACAACGTTCCTAT CCTTCTGAAGGATGCACACGTAAT	
ASGNA-F4	CTGAGTGACAATATTTTGTACTCCGGTGAGACT CTCTCTACTGGGGAATTTCTCAACTA	over-lap PCR
ASGNA-R4	AGATGCGGGAATTCCAACAAGTCCGGTGTGAG TTCCAGTAGCCCAACGATCAGTTCCGT	
ASGNA-F5	TCATTTTGGCCGCCATCTTCCTTGGTGTCATCA CACCATCTTGCCTGAGTGACAATATT	over-lap PCR
ASGNA-R5	AAGCTTAATCTTTCCAGCAGTAGGATATTTCTCT GAGGGTGGAGATGCGGGAATTCCA	
ASGNA-F6	CAACTACAAGTTACAAAATGGCTAAGGCAAGTC TCCTCATTTTGGCCGCCATCTT	over-lap PCR
ASGNA-R6	AAGTTAAAAGATCACCGGTCATTACTTTGCAGT CACAAGCTTAATCTTTCCAGCA	
ASGNA-XhoI-F	ATTCTCGAGCAACTACAAGTTACAAAATGG	pART27::ASGNA
ASGNA-XbaI-R	TTTCTAGAAAGTTAAAAGATCACCGGT	
35S-ASGNA-F1	ATCATTGCTCATCCTCACTGCT	Confirmation of transgenic plants
35S-ASGNA-R1	ATTTCTAGAAAGTTAAAAGATCACCGGT	
35S-ASGNA-R2	TCAGCGGCCGCAGATTTAGGTGACACTATAG	
